# Wuliangye *Baijiu* but not ethanol reduces cardiovascular disease risks in a zebrafish thrombosis model

**DOI:** 10.1038/s41538-022-00170-2

**Published:** 2022-12-05

**Authors:** Hui Zhu, Chaohua Lan, Dong Zhao, Ning Wang, Di Du, Huibo Luo, Huiqiang Lu, Zhifu Peng, Yumeng Wang, Zongwei Qiao, Yong Huang, Baoguo Sun

**Affiliations:** 1 Beijing Advanced Innovation Center for Food Nutrition and Human Health, Beijing Technology and Business University, 100048 Beijing, China; 2 Technology Research Center, Wuliangye Yibin Co., Ltd., 644007 Yibin, China; 3 School of Bioengineering, Sichuan University of Science and Engineering, 643000 Zigong, China; 4 Energy Sciences, ExxonMobil Technology and Engineering Company, Annandale, NJ USA; 5 Center for Drug Screening and Research, School of Geography and Environmental Engineering, Gannan Normal University, 341000 Ganzhou, China; 6 Bristol Myers Squibb, Princeton, NJ 08540 USA

**Keywords:** Cardiovascular diseases, Transcriptomics

## Abstract

Understanding how *Baijiu* facilitates blood circulation and prevents blood stasis is crucial for revealing the mechanism of *Baijiu* for cardiovascular disease (CVD) risk reduction. Here we established a zebrafish thrombosis model induced using arachidonic acid (AA) to quantitatively evaluate the antithrombotic effect of Wuliangye *Baijiu*. The prevention and reduction effects of aspirin, Wuliangye, and ethanol on thrombosis were compared using imaging and molecular characterization. Wuliangye *Baijiu* reduces thrombotic risks and oxidative stress in the AA-treated zebrafish, while ethanol with the same concentration has no similar effect. The prevention and reduction effects of Wuliangye on thrombosis are attributed to the change in the metabolic and signaling pathways related to platelet aggregation and adhesion, oxidative stress and inflammatory response.

## Introduction

Epidemiological studies showed that French has a high consumption of dietary cholesterols and saturated fat but a low mortality for coronary heart disease (CHD), which is also known as the French Paradox. This may be attributed in part to moderate alcohol intake through a hemostatic mechanism where alcohol inhibits platelet reactivity^1^. In general, moderate alcohol consumption has been found to be associated with a lower risk of cardiovascular disease (CVD)^2–4^. It was recently observed that moderate alcohol consumption reduces risks of all-cause mortality in addition to CVD events in a large population of elderly individuals^5^. Nevertheless, another study indicates that the low risk of mortality and cardiovascular event may only be associated with low levels of alcohol assumption up to approximately 105 g a week^6^. Moreover, genetic epidemiological analyses showed that the protective effects of moderate alcohol intake against stroke are largely non-causal. Alcohol consumption is found to increase blood pressure and stroke risk, but effect on the risk of myocardial infarction is not significant^7^. Besides, moderate consumption of alcohol has also been observed to be associated with increased incidence and mortality from cancer and liver cirrhosis^5,8,9^. A major limitation to the validity of the epidemiological studies is the bias due to uncertainties in exposure measurement including frequency and amount of alcohol consumption. As a result, the conclusions of the aforementioned epidemiological studies are limited to statistical correlations between alcohol consumption and CVD risk from meta-analyses of large-scale clinical data. While factors like age, sex, and smoking status can be accounted for in the statistical model, a variety of biological factors such as diet are difficult to adjust^10^. Moreover, bioactive compounds in wine such as resveratrol exert beneficial effects, but the amount of wine needed for the resveratrol to produce a significant beneficial effect is associated with a significant toxic effect from alcohol^11,12^. Thus, it is difficult to establish a direct causal relationship between wine consumption and CVD risk. Mechanistic studies based on biological models are needed to determine whether the observed epidemiologic associations are casual.

*Baijiu* is China’s national liquor with a long history of more than 2000 years. As one of the six major distilled spirits in the world, the annual output of *Baijiu* is 715.6 million liters in 2021 (released data of State Statistical Bureau of China, https://data.stats.gov.cn/easyquery.htm?cn=A01). Its unique solid mixed-culture brewing process yields rich aroma and taste as well as various bioactive compounds. Among the twelve types of aroma *Baijiu*, strong-aroma *Baijiu* has the strongest and most complex aroma and taste^13^. Wuliangye *Baijiu* is one of the most famous strong-aroma *Baijiu*, which is fermented from five different grains including sorghum, rice, glutenous rice, wheat, and corn. About 500 volatile compounds of Wuliangye *Baijiu* has been identified in more than 3000 peaks by solid-phase extraction and GC×GC-TOFMS analysis^14^. Various compounds are crucial to the flavor and quality of *Baijiu*. Many of them have been found to be beneficial to human health, including phenols, acids, pyrazines, sulfur compounds, terpenes, esters, furans, peptides, etc.^15,16^. In particular, several compounds including linoleic acid, α-Linolenic acid, ethyl linoleate, ferulic acid, guaiacol, tetramethylpyrazine, and dimethyl sulfide have been reported to resist oxidation and ameliorate atherosclerosis and prevent CVDs^17–28^.

Thrombosis is one of the leading pathological causes of morbidity and mortality in a wide range of CVDs^29^. Thrombus formation is initiated from the aggregation of circulating platelets around the damaged blood vessel walls and can lead to elevated oxidative stress^30^. Understanding how *Baijiu* facilitates blood circulation and prevents blood stasis is crucial for explaining the effect of *Baijiu* on reducing the CVD risk. Here we present a zebrafish thrombosis model induced using arachidonic acid (AA) to quantitatively evaluate the antithrombotic effect of Wuliangye *Baijiu*. We compared the prevention and reduction effects of aspirin, Wuliangye, and ethanol at the same concentration on thrombosis. We investigated how AA induces thrombosis and *Baijiu* reduces thrombosis in zebrafish using microscopy, RNA-seq, and qPCR arrays.

## Results

### Red blood cells (RBCs) and blood flow

**Fig. 1:**
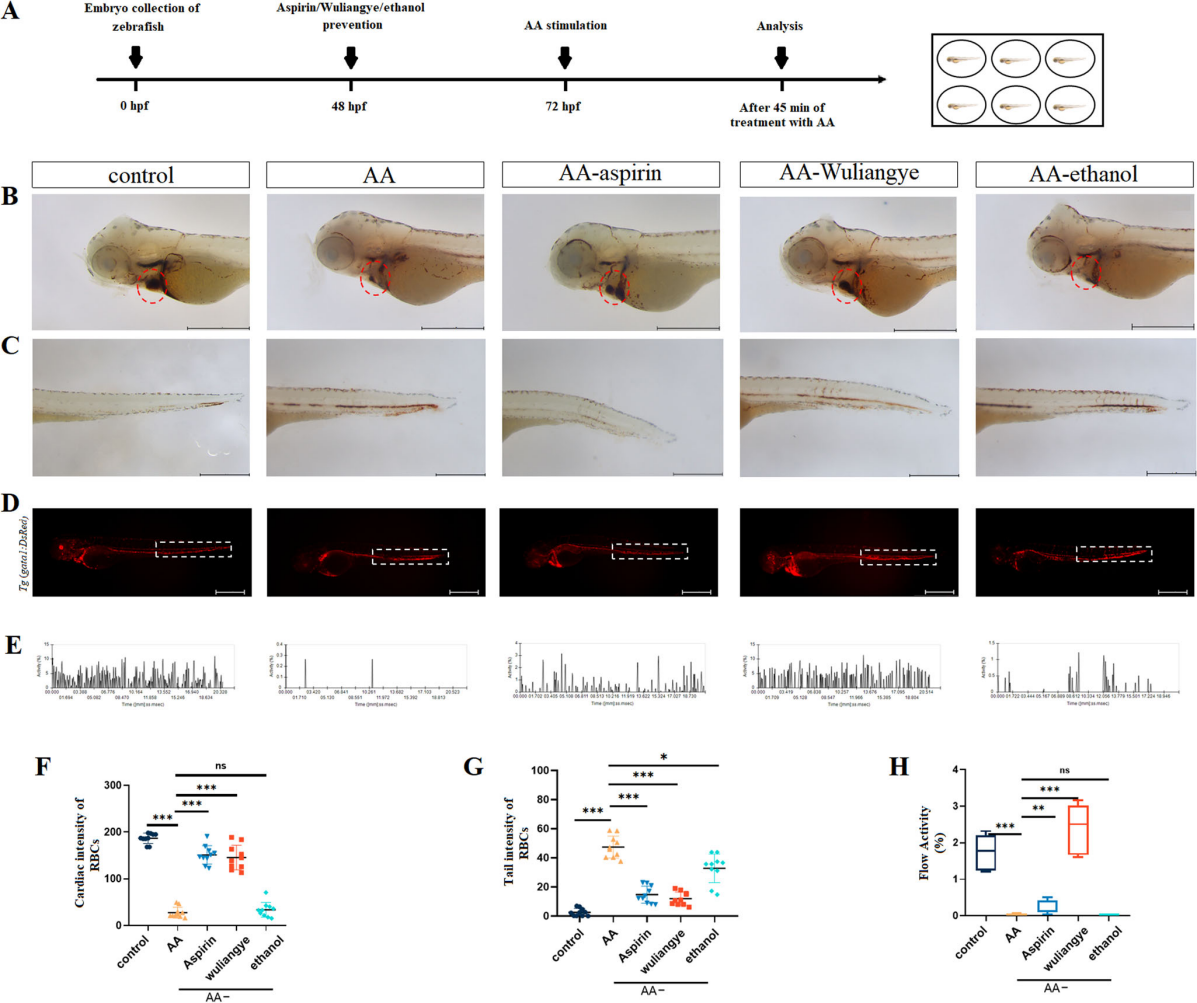
Wuliangye alleviates thrombosis in the AA-treated zebrafish. **A** Time line of aspirin/Wuliangye (0.3% ethanol, v/v), 0.3% ethanol protection and AA treatment. Representative images (**B**) and quantification (**F**) of o-dianisidine staining at the heart regions of control, AA, AA-aspirin, AA-Wuliangye, and AA-ethanol groups (*n* = 10). The heart regions are circled by red dotted line. **C**, **G** Representative images (**C**) and quantification (**G**) of o-dianisidine staining at the tail regions control, AA, aspirin, Wuliangye and ethanol treated (*n* = 10). Scale bar = 500 μm. All data are presented with mean ± standard deviation (solid bar). **D** Blood cell aggregation of Tg (gata1:DsRed) zebrafish in control group, AA group, aspirin group, Wuliangye group and ethanol group. **E** Blood flow velocity frequency diagram of zebrafish in control, AA, aspirin, Wuliangye and ethanol treated. **H** Boxplots of blood flow velocity statistics of control, AA, AA-aspirin, AA-Wuliangye and AA-ethanol groups, the boxes are outlined from the first quartile to the third quartile, the center line that goes through the box is the median, the whiskers (small lines) go from each quartile towards the minimum or maximum value. Compared with the control group; compared with the model group, ns for not significant, *P < 0.05, **P < 0.01, ***P < 0.001 (*t* test).

In order to validate whether the lack of RBCs in the heart is due to thrombus formation in the peripheral circulation or vascular defects, transgenic *Tg (gata1:DsRed)* was used to dynamically track the circulatory status of RBCs in AA-treated embryos. It can be noted that 45 mins after AA treatment in the 3dpf (days past fertilization) embryos, RBCs started to reduce in the heart and accumulate in caudal arteries and caudal veins (Fig. 1B, C). Compared to the AA group, the RBC level in the AA-aspirin-treated zebrafish was increased in the heart (*P* < 0.001) and reduced in the tail (*P* < 0.001) nearly back to the that of the control group, confirming that thrombus formation in the peripheral circulation leads to cardiac ischemia (Fig. 1 and Supplementary Videos 1–5).

Next, we investigated the effect of Wuliangye *Baijiu* and ethanol on thrombus formation. It was found that the of Wuliangye *Baijiu* with ethanol concentration (v/v) higher than 0.5% or equivalent ethanol solution significantly (*P* < 0.001) increased the zebrafish embryo malformation rate and had an impact on hatchability. When the concentration was decreased to 0.3%, there had no obvious effect on zebrafish embryo development. Therefore, Wuliangye *Baijiu* (0.3% ethanol, v/v) and 0.3% ethanol were considered for treatment. Similar to the AA-aspirin treatment, compared with the AA group, the heart RBC level of zebrafish with AA-Wuliangye treatment was significantly increased (*P* < 0.001) and the tail RBC level was significantly increased (*P* < 0.001). Those receiving AA-ethanol treatment exhibit no significant different (*P* > 0.05) compared to AA treatment, indicating ethanol does not have similar effect to Wuliangye (Fig. 1F, G).

Compared with the control group, the blood flow velocity was significantly reduced in the AA-treated zebrafish (*P* < 0.001). However, the blood flow velocity of AA-Wuliangye and AA-aspirin groups was significantly increased compared with the AA group (*P* < 0.001), but that of the AA-ethanol group exhibits no significant difference (*P* > 0.05). It is worth noting that the efficacy of Wuliangye *Baijiu* to improve blood flow in the thrombosis model is also better than aspirin.

### Oxidative stress

**Fig. 2:**
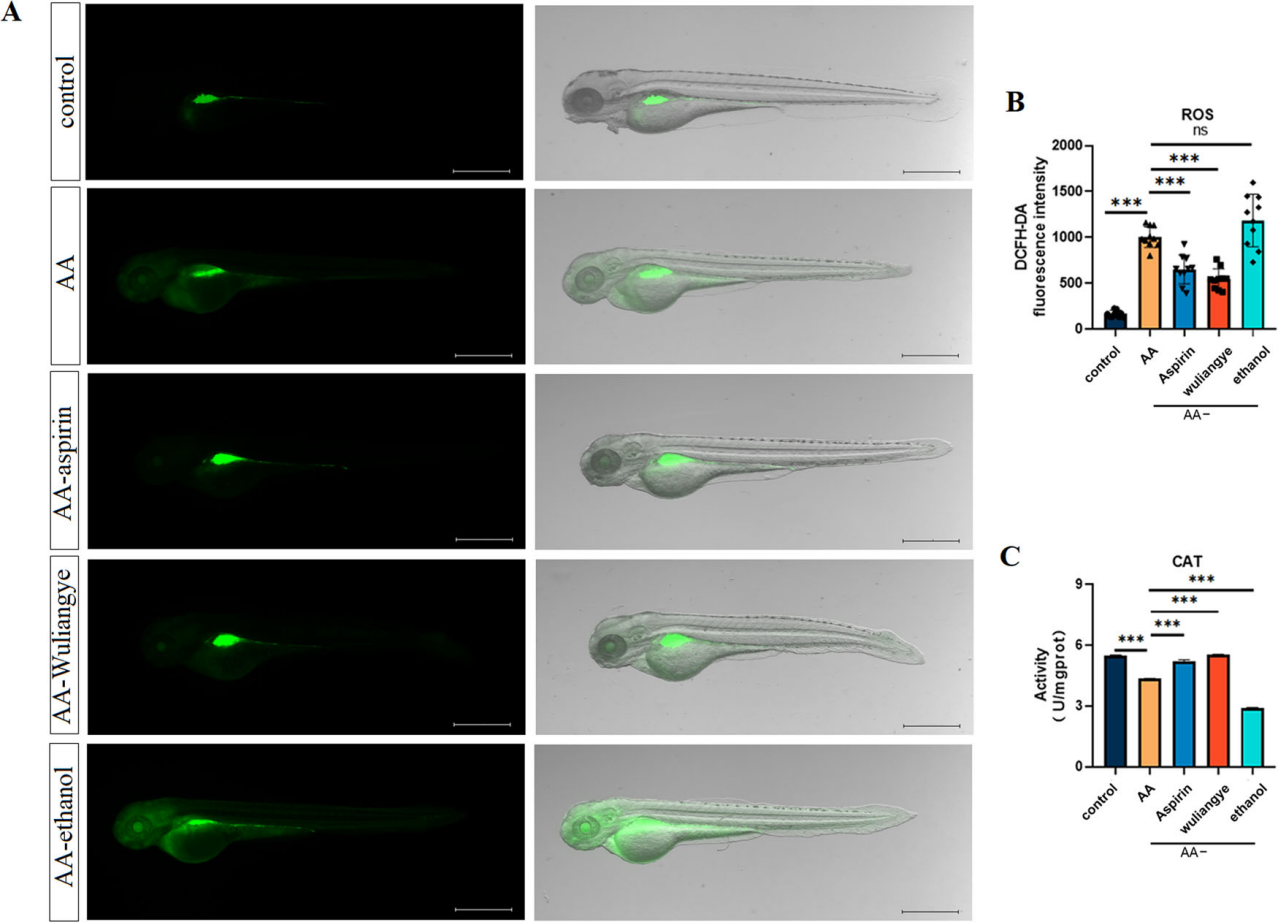
Wuliangye reduces the elevated levels of oxidative stress in the zebrafish thrombosis model. Representative images (**A**) and quantification (**B**) of the fluorescence signals of DCFH-DA staining in different treatment groups (*n* = 10). Concentration of CAT (**C**) in zebrafish in different treatment groups. Scale bar = 500 μm. Compared with the control group; compared with the model group; data are presented as mean ± standard deviation (ns for not significant, ***P < 0.001, *t* test).

We used a fluorescent H_2_O_2_ indicator DCFH-DA to monitor cellular oxidative stress. Compared with the control group, elevated DCFH-DA signal was observed in zebrafish with AA treatment, indicating that AA can lead to the increase of oxidative stress. Both Wuliangye and aspirin decreased the intensity and coverage of the DCFH-DA signal, while ethanol increased it (Fig. 2A, B). Antioxidant enzyme detection showed that CAT levels in the AA group was significantly decreased compared to the control group (*P* < 0.001) and treatment with ethanol shows no improvement. The CAT levels of the zebrafish after the AA-aspirin and AA-Wuliangye treatment were significantly increased compared with the AA group (*P* < 0.001) (Fig. 2C), indicating that Wuliangye and aspirin show significant antioxidant effects.

### RNA-Seq analysis

**Fig. 3:**
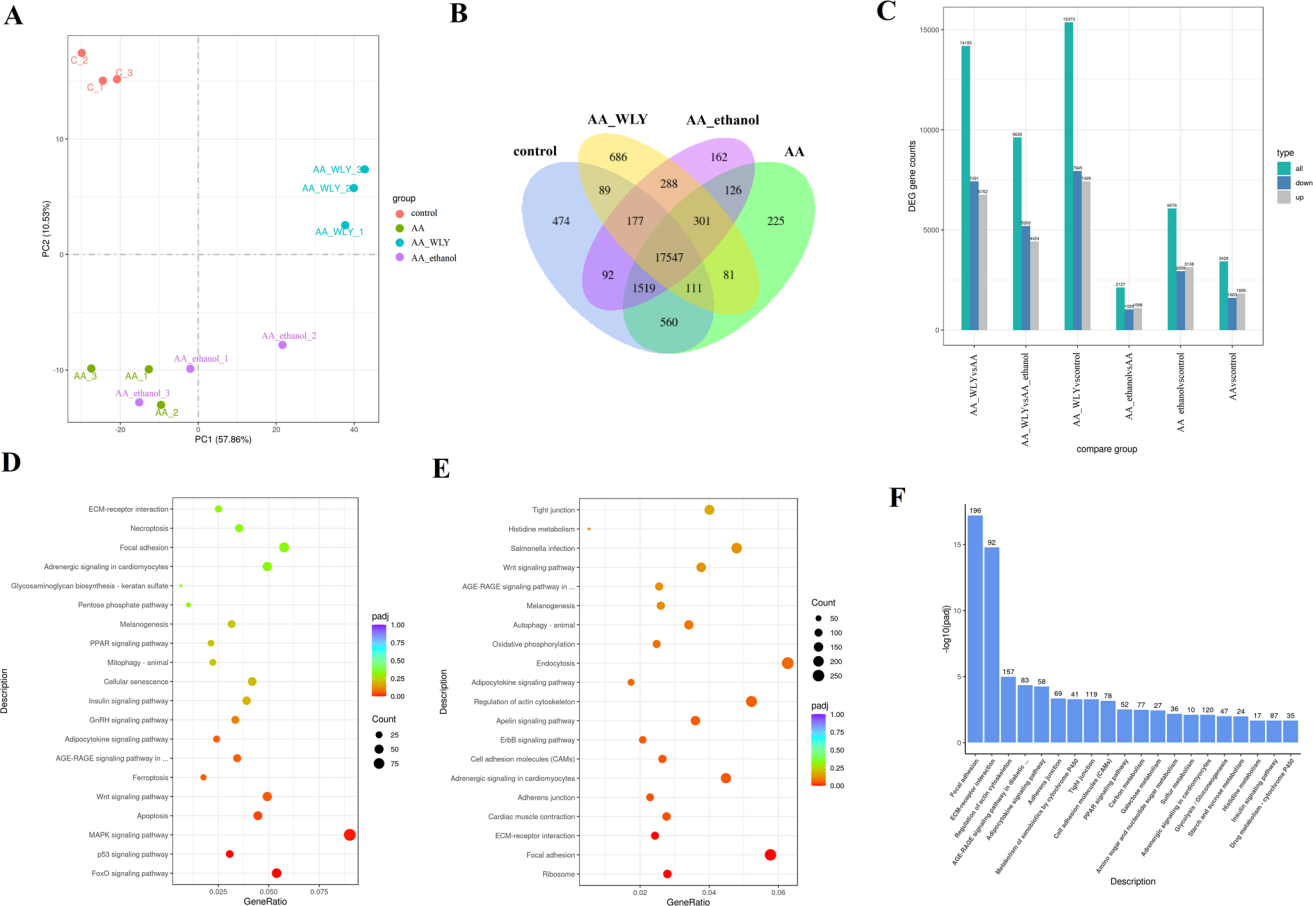
RNA-seq data analysis. **A** PCA of gene expression. **B** Venn diagram of gene counts expressed. **C** Significant DEG counts. **D** The KEGG pathway enrichment of DEGs between AA and control. **E** The KEGG pathway enrichment of DEGs between AA and AA-Wuliangye. **F** Pathway Enrichment Analysis of downregulated DEGs between AA and AA-Wuliangye groups.

We performed transcriptomic analysis of the samples using RNA-seq (Supplementary Table 2). The principal component analysis (PCA) indicated that the first two principal components of the gene expression levels of the different groups exhibit distinct individual clusters (Fig. 3A).

The genes with a reads per kilobases per million (RPKM) ratio greater than twofold were defined as DEGs. Significant DEGs, including upregulated or downregulated genes, were identified by DEGseq (Fig. 3B). Compared with the AA group, 14193 DEGs, including 6762 upregulated and 7431 downregulated genes, were identified in the AA-Wuliangye group, 2127 DEGs, including 1098 upregulated and 1029 downregulated genes, were identified in the AA-ethanol group (Fig. 3C).

To further reveal the role of Wuliangye in the prevention and reduction effects against AA-induced thrombosis, we performed KEGG pathway analysis on pairs of individual groups. Compared with the control group, most of the DEGs were enriched to inflammation and oxidative stress response in AA group zebrafish (Fig. 3D). DEGs between AA and AA-Wuliangye groups were associated with platelet aggregation and adhesion, inflammatory or immune response, and oxidative stress reaction (Fig. 3E). Significant decreases in the expression of key genes involved in focal adhesion, ECM-receptor interaction, AGE-RAGE signaling pathway, adherens junction, cell adhesion molecules (CAMs), PPAR signaling pathway, *etc*. This confirms that Wuliangye treatment reduces thrombosis by regulating inflammation and oxidative stress related pathways (Fig. 3F).

### qRT-PCR

**Fig. 4:**
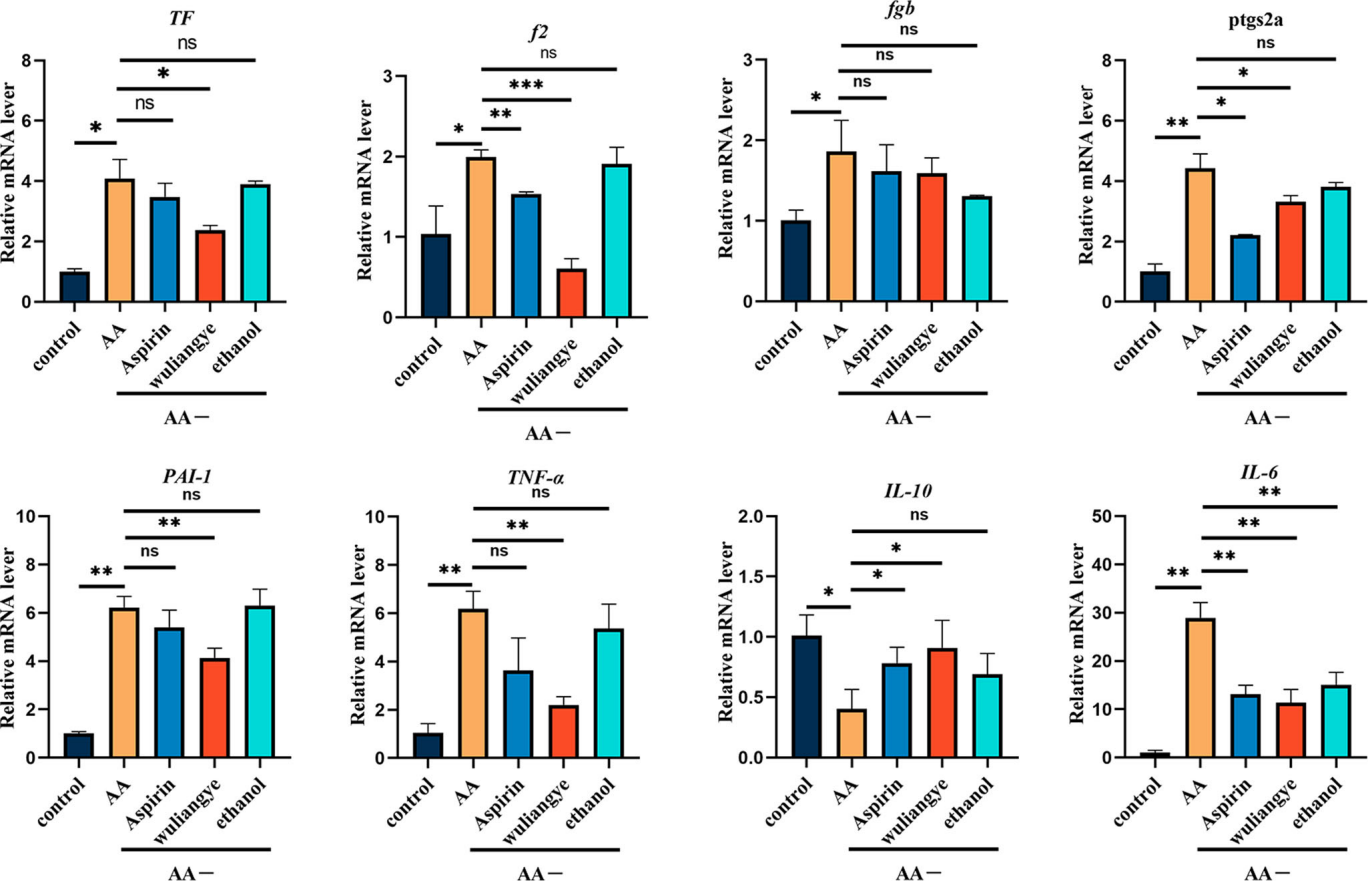
Changes of thrombus-related gene expression. Compared with the control group; compared with the model group; data are presented as mean ± standard deviation (ns for not significant, *P < 0.05, **P < 0.01, ***P < 0.001, *t* test).

We used qPCR arrays to detect the expression of a focused panel of coagulation and platelet activation factors including tissue factor (TF), coagulation factor II (f2), fibrinogen β chain (fgb), prostaglandin peroxide synthase 2A (ptgs2a), plasma plasminogen activator inhibitor PAI-1, tumor necrosis factor (TNF-α), interleukin-10 (IL-10), and interleukin-6 (IL-6) (Fig. 4). Compared to the control group, the transcriptional levels of all the aforementioned genes are significantly changed in the AA group. The changes are found to be largely mitigated using either aspirin or Wuliangye treatment. In particular, Wuliangye significantly mitigated the change in the expression levels of 7 genes including TF, f2, ptgs2a, PAI-1, TNF-α, IL-10, and IL-6. Aspirin significantly mitigated the change in the expression levels of 4 genes including f2, ptgs2a, IL-10, and IL-6, while ethanol only mitigated the change for IL-6.

## Discussion

Thrombosis is a pathological result of atherosclerosis and a major cause of death from coronary heart disease. Thrombi are mainly formed through the accumulation of blood in blood vessels, which occurs in a complex and gradual manner. Thrombosis can occur at artery, vein, and micro-vessels. Among them, arterial thrombus is mainly composed of platelets and leukocytes and venous thrombus is mainly composed of fibrin and red blood cells. Despite different compositions, they may have similar pathogenic mechanisms, such as inflammation, hypercoagulable state and endothelial injury.

Zebrafish share 87% genome with humans and have similar coagulation factors and platelet receptors^31^. Therefore, zebrafish are widely used as a thrombosis model to study thrombosis mechanisms and evaluate the efficacy of antithrombotic drugs^32–35^. As we know, this study evaluates the causal association between *Baijiu* consumption and CVD risk by using zebrafish model. Studies have shown that arachidonic acid (AA) can directly activate platelets, induce platelet aggregation, and form thrombi. AA-induced thrombosis is thought to be representative of both venous and arterial thrombi^35^. In this study, a robust thrombosis model was established by optimizing the induction conditions, and the antithrombotic effects of Wuliangye and pure ethanol were evaluated. We found that Wuliangye *Baijiu* (0.3% ethanol, v/v) can prevent and reduce thrombosis, while ethanol at the same concentration does not, indicating that the bioactive compounds in Wuliangye *Baijiu* may play an important role in the antithrombotic process.

Baijiu as a natural fermented food, has complex bioactive compounds. The strong-aroma *Baijiu* Wuliangye is rich in esters, phenolic compounds, sulfur-containing compounds, pyrazines and terpenes^14^. The antioxidant, anti-inflammatory, antithrombotic activities and vasodilating effects of these compounds have been investigated in depth^17–28^. Unlike aspirin and other monomeric drugs, which mainly act on specific receptor /pathways, various bioactive compounds in Baijiu have different action mechanisms. Even specific compound may be involved in multiple pathways. For example, although phenols, pyrazines, unsaturated fatty acid esters all have antioxidant and antithrombotic activity, different compounds focus on regulating different genes/pathways. Pyrazines significantly regulate arachidonic acid metabolism, while unsaturated fatty acid esters mainly affect linoleic acid metabolism pathway. In RNA-seq results, a total of 14193 DEGs were identified between AA-Wuliangye and AA group, which was more than 2/3 of identified genes. The number of DEGs between AA-Wuliangye and other groups were all extremely high. The results indicated that the effect of Wuliangye on prevention of thrombosis may be attributed to the synergistic effect of multiple bioactive compounds. The prevention and reduction effects of Wuliangye on thrombosis are attributed to regulation of key genes and pathways related to oxidative stress, platelet aggregation and adhesion and inflammatory response.

Oxidative stress and lipid peroxidation are well studied risk factors for hypercoagulability and thrombosis^36,37^. In the vascular system, oxidative stress helps regulate vasoconstriction, platelet aggregation, angiogenesis, and many other physiological processes. Oxidative stress is involved in the pathological process of thrombotic diseases, such as arteriosclerosis, stroke, myocardial infarction^38^ AA can accelerate production of reactive oxygen species, leading to endothelial dysfunction and coagulation disorders that cause oxidative stress and lipid peroxidation, and ultimately thrombi^39^. Therefore, we evaluated the antioxidant effect of Wuliangye by detecting reactive oxygen species (ROS) and catalase (CAT) in zebrafish tissues. The results showed that ROS in the AA group and the ethanol group were significantly increased, and CAT levels were significantly decreased. This confirms that AA can induce platelet aggregation in zebrafish by elevating oxidative stress in vivo. Wuliangye can significantly reduce oxidative stress and platelet aggregation, while ethanol does not exhibit similar effect.

Thrombosis results from complex interactions between coagulation factors and cellular components of blood^40^. Platelets have a variety of receptors that sense activating signals including thrombin, ADP, epinephrine, as well as the certain receptors for ECM proteins^41^. These diverse receptors trigger intracellular signaling pathways that contribute to the adhesion and aggregation of activated platelets. As a result, pharmacological control of platelet activation becomes the major strategy for the prevention of arterial thrombosis^42^. Our pathway analysis indicated that the Wuliangye treatment downregulated the expression of a large number of genes involved in multiple pathways related to platelet activation, including focal adhesion, ECM-receptor interaction, AGE-RAGE signaling pathway, adherens junction, cell adhesion molecules (CAMs). This confirms that Wuliangye reduces platelet adhesion, activation, and aggregation to regulate the coagulation cascade through these pathways.

Tissue factor (TF), a transmembrane protein, is the main promoter of the coagulation cascade. Upon vascular injuries, TF around the blood vessel is exposed to the blood and forms TF:FVIIa complex. This leads to the activation of FX and FIX, and subsequent generation of thrombin, fibrin deposition, and platelet activation^43,44^. Under pathological conditions, TF is expressed on the membrane of nearly all blood cells and vascular endothelial cells, which can trigger coagulation cascade leading to disseminated intravascular coagulation or thrombosis. Fibrinogen, including fga, fgb and fgg polypeptides^45^, is the precursor of the fibrin network and one of the most abundant coagulation factors. It can be rapidly converted into fibrin monomers and insoluble fibrin polymers, which play an important role in the coagulation cascade and thrombus formation^46–48^. In addition, elevated fibrinogen (fga) and thrombin (f2) levels can induce hypercoagulability and thrombosis^49^. Our QPCR array analysis showed that AA can significantly increase the expression of coagulation cascade reaction factors including TF, f2, and fgb. Wuliangye intervention can significantly inhibit the expression of these factors, but ethanol does not exhibit similar effect. This confirms that the bioactive compounds in Wuliangye are related to the inhibition of coagulation cascade.

Both pro-inflammatory and anti-inflammatory factors have important regulatory roles in thrombosis^50^. For example, inflammation-related cytokines including interleukin-6 (IL-6) and tumor necrosis factor-alpha (TNFα), are associated with an increased risk of venous thrombosis^51^. Fibrin and its degradation products can regulate the inflammatory response. Fibrin can promote the migration of leukocytes and directly interact with peripheral blood mononuclear cells through CD11b/cd18 integrins, resulting in elevated activities of TNFα, IL-6 and other inflammatory cytokines^52^. Plasminogen activator inhibitor (PAI-1), secreted by platelet α–granules, is a major inhibitor of plasma fibrinolytic activity. PAI-1 is produced at the inflammation site after tissue injury and plays a regulatory role during local inflammation^53^. Thrombolytic resistance is usually found to be inversely correlated to PAI-1^54^. ptgs2b is the homologous gene of mammalian prostaglandin endoperoxide synthase 2 (ptgs2), which plays a key role in the inflammatory response^55,56^. Our qPCR results showed that AA pretreatment increased the expression of TNF-α, IL-6, ptgs2b and PAI-1 in zebrafish but Wuliangye reduced the levels of those AA-induced inflammatory mediators. The expression of IL-10, an anti-inflammatory factor, was significantly reduced after AA treatment, but Wuliangye treatment increased its expression. On the contrary, ethanol exhibits no significant effect to the inflammatory mediators except IL-6. This indicates that the bioactive compounds in Wuliangye may improve inflammatory response.

A zebrafish thrombosis model was established to investigate the effects of Wuliangye in thrombus formation. The study showed that Wuliangye *Baijiu* reduces thrombotic risks and oxidative stress in the AA-treated zebrafish, while ethanol with the same concentration has no similar effect. RNA-seq and qPCR analyses revealed that the effect of Wuliangye on thrombosis is attributed to the mitigation of platelet aggregation and adhesion, oxidative stress and inflammatory response. Our findings provide important insights into the molecular mechanisms of the prevention and reduction effects of Wuliangye on thrombosis. Further studies will be needed to confirm the specific compounds in Wuliangye that contributes to the beneficial effects.

## Methods

### Chemicals and reagents

Arachidonic acid (AA, CAS 506-32-1) was purchased from Shanghai Yuanye Biotechnology Co, LTD. Aspirin was purchased from MedChemExpress. Wuliangye was provided from Wuliangye Yibin Co., Ltd. (https://www.wuliangye.com.cn/). Anhydrous ethanol and Trizol reagent were purchased from Sangon Biotech (Shanghai) Co, Ltd. The reverse transcription kit, CAT, ROS and other detection kits were purchased from Nanchang Excellence Biotechnology Co, LTD. TransStart Green qPCR SuperMix (AQ141-02) was obtained from Jiangxi Biyou Technology Co, LTD.

### Animal care ethics

All zebrafish experiments were conducted according to the guidelines of Animal Ethics Committee of the Laboratory Animal Center, Gannan Normal University, Jiangxi.

### Zebrafish husbandry and embryo collection

Transgenic *Tg (gata1:DsRed)* zebrafish with enhanced expression of red fluorescent protein (DsRed) in the blood corpuscle were purchased from China Zebrafish Resource Center. These zebrafish were kept in flow-through tanks with aerated freshwater at 28 ± 0.5°C under a 14/10 h light/dark cycle and fed with freshly hatched brine shrimp according to the Institutional Animal Care and Committee protocols.

In order to collect embryos, males and females were placed in mating tank at the ratio of 1:1 to 1:2 and separated by a barrier. The next morning, the barrier was removed and the females started to lay eggs. The embryos were collected within half an hour. After removing dead and unfertilized eggs, feces, and other debris, the viable embryos were washed several times with egg water, and then incubated at 28.5°C for 24 h. Melanin production was inhibited by adding 1-phenyl-2-thiourea (PTU).

### Zebrafish thrombosis modeling and drug treatment

In this study, AA was used to induce thrombus formation and aspirin, a clinically effective drug for thrombosis, was used as a positive control^57,58^. According to previous studies, different concentrations of AA including 30, 40, 50, and 60 μM were tested for 30, 40, 50, 60, and 90 min, respectively^56,59,60^. The thrombosis model was optimized to be under 30 μM concentration for 45 min in our study. In order to further screen the optimal treatment concentration, 48 h-post- fertilization (hpf) zebrafish embryos were exposed to Wuliangye *Baijiu* and ethanol solutions with ethanol concentration (v/v) at 0.1, 0.3, 0.5, 0.7, 0.9, and 1.1% for 24 h to investigate the hatchability and developmental status.

For the treatment group, starting from the 48 hpf, embryos were incubated in 22.5 mg/L aspirin, optimum wuliangye and ethanol, respectively, until samples were collected for measurements. At 72 hpf, the thrombosis was induced using 30 μM AA treatment for 45 min (Fig. 1A). Since AA was dissolved with 0.075% DMSO, 0.075% DMSO was used as a blank control. As a result, 5 different groups including control, AA, AA-aspirin, AA-Wuliangye, and AA-ethanol were present in the study.

### o-Dianisidine staining

Zebrafish has a simple blood circulation system from heart to dorsal aorta and caudal vein and then back to heart^61^. Therefore, a thrombus formed in the zebrafish is expected to increase red blood cell (RBC) level in the caudal vein and decrease RBC level in the heart^61^. After treatment, the embryos from each group were stained with o-dianisidine, which was used to detect RBC level, and randomly selected for quantitative analysis of thrombosis through imaging^59^.

### Analysis of blood flow and RBC aggregation

In order to record blood flow videos, the embryos were fixed in 1% of low melting agarose without anesthesia. Blood flow velocity was calculated using DanioScope1. The playback speed was slowed down to 6 frames per second in Supplementary Videos. Blood flow of at least 10 zebrafish embryos was analyzed in each group. Moreover, RBC aggregation of Transgenic zebrafish *Tg (gata1:DsRed)* was analyzed. The embryos were fixed in 1% of low melting agarose with 0.4% tricaine anesthesia for microscopy imaging. Images were taken under a fluorescence microscope Leica M205 FA stereoscopic microscope (Germany).

### Oxidative stress analysis

DCFH-DA, a fluorescent indicator of H_2_O_2_ or other ROS, was used as a marker for cellular oxidative stress^62^. The embryos were incubated in 1000× diluted DCFH-DA at 28.5 °C for 20 min in the dark and imaged under a fluorescence microscope (Leica M205 FA stereo microscope, Germany). Fluorescence intensity of ROS staining was calculated using Image J (NIH, USA).

Catalase (CAT) is an enzyme that catalyzes the decomposition of hydrogen peroxide into oxygen and water, which represents the antioxidant capacity of the organism. CAT activity was measured using suitable kits according to the kit instructions. Total protein level was quantified using Coomassie Brilliant Blue staining. Absorbance was measured using a multifunctional micrometer (PerkinElmer Victor nivo, USA) with each sample measured three times. Absorbance was normalized to total protein levels to assess the effect of AA on embryonic oxidative stress and to compare changes of oxidative stress in AA zebrafish pretreated with aspirin, Wuliangye and ethanol at the same concentration. 50 embryos with 3 biological replicates were collected from each group.

### RNA isolation to construct library

Thirty embryos were collected from each group at 3 days post-fertilization (dpf) and washed five times with phosphoric acid buffer. RNA was extracted and quantified using Trizol reagent (Invitrogen, Carlsbad, USA). After determining the RNA quality and quantity, the libraries were sequenced on an Illumina HiSeq X Ten platform. Then 150 bp paired-end reads were obtained.

### RNA-Seq analyses

Transcript assembly and functional assignment were performed as described earlier^63^. Differential expression analyses were performed between groups with duplicates using the DESeq2 R package (1.16.1). Gene Ontology (GO) analysis of differentially expressed genes (DEGs) was performed using the clusterProfiler R package with gene length bias corrected. GO terms with corrected *P* value < 0.05 were considered significantly enriched by DEGs. Statistical enrichment of DEGs in KEGG pathways was evaluated in clusterProfiler R package.

### RNA extraction and qRT-PCR

50 embryos were collected from each treatment group and washed five times with phosphoric acid buffer. The embryos were homogenized using the TriZol reagent (Invitrogen) to extract the RNA, which was then reverse-transcribed to cDNA using a Prime Script® RT reagent kit. Real-time PCR was performed using the Applied Biosystems Step-One-plus real-time PCR system (Analytic Jena, Germany/qTower 3G) with the SYBR Green detection kit and *β*-actin as the internal control. The primer sequences were shown in Supplementary Table 1. Each sample was tested in triplicates.

### Statistical analyses

Statistical analyses including one-way ANOVA and Student’s *t* test were performed using the GraphPad Prism 5.0 Software. All values were shown as mean ± standard deviation. The resulted *P* values were annotated as follows: *P < 0.00.5, **P < 0.01, and ***P < 0.001 unless otherwise noted.

### Reporting summary

Further information on research design is available in the Nature Research Reporting Summary linked to this article.

## Supplementary information

Supplementary Material

Reporting Summary

Supplemental video 1 Blood flow of zebrafish in control group.

Supplemental video 2 Blood flow of zebrafish in AA group.

Supplemental video 3 Blood flow of zebrafish in AA-aspirin group.

Supplemental video 4 Blood flow of zebrafish in AA-Wuliangye group.

Supplemental video 5 Blood flow of zebrafish in AA-ethanol group.

## Data Availability

The raw sequence data reported in this paper have been deposited in the Genome Sequence Archive (Genomics, Proteomics & Bioinformatics 2017) in National Genomics Data Center (Nucleic Acids Res 2021), China National Center for Bioinformation/Beijing Institute of Genomics, Chinese Academy of Sciences, under accession number CRA006632 that are publicly accessible at https://bigd.big.ac.cn/gsa.
