# Supplementary material for: Wuliangye Baijiu but not ethanol reduces cardiovascular disease risks in a zebrafish thrombosis model
Source: NPJ Sci Food. 2022 Dec 5;6:55. doi: 10.1038/s41538-022-00170-2 (PMC9723178; doi:10.1038/s41538-022-00170-2)
Supplement: Supplementary file 1 — Supplementary Material [file 41538_2022_170_MOESM1_ESM.pdf]

Supplementary Table 1 Primers sequences for qRT-PCR

Gene	Forward Sequence	Reverse Sequence
β-actin	5′-AGCACGGTATTGTGACTAACTG-3′	5′-TCGAACATGATCTGTGTCATC-3′
TF	5′-GCCATACTGCATCAGGACGA -3′	5′- TCCTTCGTTTTCCGCTCTCC-3′
f2	5′- TGGAAGGAAAACCTGAACCG -3′	5′- TGAGGTCCAAGACTCCCGAA -3′
fgb	5′- CTCAGAGAGCCAAGTGCCAA -3′	5′- CCAACGCCTGCCAAAATCAA -3′
ptgs2b	5′- CAACGCCATCTTTGGGGAGA -3′	5′- CATAGGACATGGCCCGTTGA -3′
PAI-1	5′- GGGCTACAGGTGTTGCTGA-3′	5′-CACGCCATCCTTAGACACGA -3′
TNF-α	5′- GCTGGATCTTCAAAGTCGGGTGTA-3′	5′- TGTGAGTCTCAGCACACTTCCATC-3′
IL-10	5′-AGCACTCCACAACCCCAATC-3′	5′-AGCAAATCAAGCTCCCCCATA-3′
IL-6	5′-TCAGCACTCCTCTCCTCAAA-3′	5′-ATCCATCTCTCCGTCTCTCAC-3′

Supplementary Table 2 Summary of RNA-sequencing data

sample	raw_reads	raw_bases	clean_reads	clean_bases	error_rate	Q20	Q30	GC_pct
C_1	47764946	7.16G	45703968	6.86G	0.03	97.21	92.45	46.49
C_2	55267284	8.29G	52918028	7.94G	0.03	97.31	92.69	47.06
C_3	46083064	6.91G	43258704	6.49G	0.03	97.38	92.73	46.53
AA_1	47780844	7.17G	45482678	6.82G	0.03	97.23	92.5	46.42
AA_2	47821442	7.17G	45896972	6.88G	0.03	97	91.99	46.5
AA_3	49216932	7.38G	46958978	7.04G	0.03	97.33	92.73	47.17
AA_WLY_1	43546888	6.53G	41241660	6.19G	0.03	96.6	91.04	44.77
AA_WLY_2	45853276	6.88G	43561892	6.53G	0.03	96.92	91.87	44.49
AA_WLY_3	43192262	6.48G	41119010	6.17G	0.03	96.97	91.95	44.54
AA_ethanol_1	44169188	6.63G	42812960	6.42G	0.03	96.9	91.81	45.84
AA_ ethanol _2	40733108	6.11G	38835560	5.83G	0.03	96.58	91.19	45.51
AA_ ethanol _3	41445666	6.22G	39758634	5.96G	0.03	97.35	92.71	46.9

Note: raw_ Reads: the number of reads in the raw data, clean_ Reads: the number of filtered reads of the raw data, clean_ Bases: base number of filtered raw data (clean base = clean reads * 150bp), error_ Rate: overall sequencing error rate of data, Q20: percentage of bases with phred value greater than 20 in total bases, Q30: percentage of bases with phred value greater than 30 in total bases, GC_ PCT: percentage of G and C in four bases of clean reads.

Supplemental video 1 Blood flow of zebrafish in control group.

Supplemental video 2 Blood flow of zebrafish in AA group.

Supplemental video 3 Blood flow of zebrafish in AA-aspirin group.

Supplemental video 4 Blood flow of zebrafish in AA-Wuliangye group.

Supplemental video 5 Blood flow of zebrafish in AA-ethanol group.
